# WikiLink: An Encyclopedia-Based Semantic Network for Design Creativity

**DOI:** 10.3390/jintelligence10040103

**Published:** 2022-11-14

**Authors:** Haoyu Zuo, Qianzhi Jing, Tianqi Song, Lingyun Sun, Peter Childs, Liuqing Chen

**Affiliations:** 1 Dyson School of Design Engineering, Imperial College London, London SW7 2AZ, UK; 2 Department of Computer Science and Technology, Zhejiang University, Hangzhou 310030, China; 3 Singapore Innovation and AI Joint Research Lab, Zhejiang University, Hangzhou 310030, China

**Keywords:** creativity, design, concept generation, data-driven-design, knowledge discovery, semantic network

## Abstract

Data-driven design is a process to reuse data sources and provide valuable information to provoke creative ideas in the stages of design. However, existing semantic networks for design creativity are built on data sources restricted to technological and scientific information. Existing studies build the edges of a semantic network on statistical or semantic relationships, which are less likely to make full use of the benefits from both types of relationships and discover implicit knowledge for design creativity. Therefore, to overcome the gaps, we constructed WikiLink, a semantic network based on Wikipedia, which is an integrated source of general knowledge and specific knowledge, with broad coverage of disciplines. The weight in WikiLink fuses both the statistic and semantic weights between concepts instead of simply one type of weight, and four algorithms are developed for inspiring new ideas. Evaluation experiments are undertaken, and the results show that the network is characterised by high coverage of terms, relationships and disciplines, which demonstrates and supports the network’s effectiveness and usefulness. A demonstration and case study results indicate that WikiLink can serve as an idea generation tool for creativity in conceptual design. The source code of WikiLink and the backend data are provided open-source for more users to explore and develop.

## 1. Introduction

Design is a ubiquitous process that occurs throughout a variety of fields. Conceptual design is the early stage of design where an initial idea is formulated (Childs 2013). The progression of conceptual design development requires a designer to fully utilize their creativity capability and existing knowledge. In other words, the creative attributes of conceptual design depend highly on a designer’s ability to master, apply and utilize human-centred, scientific and technological knowledge according to the design problem to provoke design creativity. Researchers have utilized a large amount of imagery data or textual data available on the Internet to provide design intuition for novel ideas. This imposes a heavy challenge (Hao et al. 2014) for designers on how to effectively discover and acquire pertinent knowledge and information to promote design creativity.

With the advent of big data, semantic networks can represent associations well between ontology-based knowledge, making it easier and more intuitive to discover implicit knowledge for creativity in the early stage of design. The highly diverse nature of design suggests that design creativity can benefit from a multiplicity of distinct data. However, existing semantic networks for design creativity are built on data sources restricted to technological and scientific knowledge. Existing studies build the edges of a semantic network either with statistical or semantic relationships, which are less likely to make full use of the benefits from both types of relationships and discover implicit knowledge for design creativity.

To address the challenges highlighted, this study proposed an encyclopedia-based network called WikiLink for the creativity in the early stage of design. The source code of WikiLink is published on https://github.com/zjud3/WikiLink, accessed on 2 November 2022. The main contributions of this paper can be summarized as follows:

1. A semantic network for design creativity is constructed. Wikipedia is applied as the data source for the semantic network, which contains information from a wide range of fields and expands the data to a new boundary.
2. A combined weight is introduced for the relationship in the semantic network. The combined weight mixes the statistical relationship and semantic relationship which better captures the implicit connection between concepts for design creativity. Four algorithms are further developed for design which enables the retrieval with different levels and manners.
3. The constructed semantic network for design creativity is further developed as a tool. An evaluation and demonstration for the tool are subsequently conducted. The results show that WikiLink can effectively provide design stimuli for idea generation.

The paper is organised as follows: Section 2 describes the state of knowledge and background for the research, and Section 3 introduces the process of constructing WikiLink. Section 4 presents the experimentation including the results on coverage of concepts, coverage of relationships, coverage of disciplines and term-to-term relationships. Section 5 demonstrates the use of four functions in WikiLink and presents a design case with WikiLink. Finally, Section 6 concludes with limitations and suggestions for further research directions. It should be noted that the concepts “design” and “design creativity” in this paper refer to early stage design only.

## 2. Related Work

### 2.1. Creativity in Design and Idea Generation

Design can be regarded as the process of conceiving, developing and realising products, artefacts, processes, systems, services, platforms and experiences with the aim of fulfilling identified or perceived needs or desires, typically working within defined or negotiated constraints (Childs 2013). The standard definition of creativity is summarized as: the ability to produce original and useful products (Runco and Jaeger 2012), a definition that applies to all domains of creativity, from humor to the culinary arts and science to inventions. The creativity in design is the progress of creating innovative design, which needs the designer to fully utilize their ability to generate a design idea. Normally, creativity in the design process can benefit from considering as many ideas as possible (Liu et al. 2003). Ideas, especially creative ideas, are an essential part of the design creativity process (Han et al. 2018a, 2018b).

Much research has endeavored to propose novel approaches for idea generation (Childs et al. 2022). The diverse idea generation techniques include brainstorming (Osborn 1953), brainwriting (Geschka 1983), checklists (Ivanov and Cyr 2014) and synectics (VanGundy 1988). Recently, data-driven approaches have attracted researchers’ attention. In the process of design creativity, data-driven approaches attempt to uncover useful design knowledge from huge, unstructured, heterogeneous and highly contextualized data resources (Cheong et al. 2017; Luo et al. 2021; Shi et al. 2017). Researchers emphasize the importance of generating creative ideas in the design creativity process from big data (Howard et al. 2008; Kwon et al. 2018) and further indicate that creative ideas can originate from diverse existing knowledge and defined associations.

### 2.2. Semantic Network

Boden (1998) suggest that AI/computer techniques can be used to enhance creativity. A semantic network is a graph with nodes representing concepts or individual objects and edges representing relationships or associations among concepts (Sowa 2014). By employing the notion and theory of a network, we construct a network representing a thinking space as a model of the concept generation process and analyze its structure in order to find thinking patterns. The semantic network can represent the thinking space as a model of the concept generation process (Yamamoto et al. 2009) and help integrate and migrate valuable, unstructured data into systematic robust knowledge for design creativity (Georgiev and Georgiev 2018; Gorti et al. 1998; Rezgui et al. 2011).

When design work is completed, a great quantity of data and information are usually accumulated and reported afterwards (Ackoff 1989) in the format of proceedings, literature, patents or public reports. These pieces of recorded information are expected to be transformed into design knowledge, which is expected to be reused for new design tasks to speed up more design work. When considering knowledge reuse, common knowledge sources generally include research papers, patent documents and encyclopedias.

Academic papers and patents usually represent original research outcomes or totally new inventions, which contain rich scientific and technological knowledge. Several attempts (Fu et al. 2013; He et al. 2019; McCaffrey and Spector 2018; Munoz and Tucker 2016; Sarica et al. 2020; Shi et al. 2017) have been made to apply the academic paper and patents to a design creativity task. However, one of the major limitations is that patents and scientific literature are restricted to only technological and scientific knowledge (Ernst 2003; Furukawa et al. 2015; Li et al. 2019; Shibata et al. 2008), while the nature of design tasks is of high diversity and complexity, with broad coverage of disciplines. To address the issue, an encyclopedia can be applied for design creativity since the most notable advantage of an encyclopedia is that it contains information from a wide range of fields and can expand the design knowledge coverage to a wider boundary compared with paper and patents (Kwon et al. 2018).

### 2.3. Semantic Network for design Creativity

The main roles of semantic networks in engineering design studies include facilitating knowledge retrieval, association and reasoning. Knowledge retrieval in design engineering is retrieving the related entities and relationships from semantic networks to aid design related applications, such as query recommendation (Han et al. 2018b; Sarica et al. 2019; Siddharth and Chakrabarti 2018), and knowledge discovery (Chen and Krishnamurthy 2020; Geum and Park 2016; Goel et al. 2012; Hu et al. 2017; Linsey et al. 2012; Siddharth and Chakrabarti 2018; Vattam et al. 2011). By retrieving, the result could widen the results of the existing search queries and explore the knowledge based on semantic relationships (semantic or statistical). Knowledge association, or link prediction, is to predict and connect the unlinked entities in an existing semantic network (Han et al. 2018a; Taura et al. 2012). Reasoning in design engineering is using the semantic networks to support various applications, such as helping computers understand the meaning of words (Geum and Park 2016; Hu et al. 2017; Liu et al. 2020; Sosa et al. 2014), classifying knowledge (Goucher-Lambert and Cagan 2019; Yuan and Hsieh 2015) and idea generation (Georgiev et al. 2017; Han et al. 2018b).

Most of the design-engineering-related semantic networks mentioned are based on common-sense knowledge, such as WordNet (Fellbaum 2010), ConceptNet (Speer et al. 2017), Wikidata (Vrandečić and Krötzsch 2014), DBpedia (Auer et al. 2007) and Yago (Suchanek et al. 2007). WordNet is an expert-developed English database, which is interlinked by semantic relations including synonyms, hyponyms, and meronyms as an extension of a dictionary and thesaurus. ConceptNet is a freely available semantic network aiming at helping computers understand the meanings of words that people use. The words are connected via common-sense relations, such as “IsA”, “HasA” and “HasProperty”. Wikidata, DBpedia and YAGO are other large-scale general knowledge semantic networks (or knowledge graphs), which consist of entities and relationships from WordNet or Wikipedia via an unsupervised approach. These general semantic networks were first developed for artificial intelligence tasks such as machine translation and natural language understanding (Sowa 2014). When employed in design related tasks, they are designed as the backend knowledge to computational tools for design idea generation and analysis (Bae et al. 2020; Georgiev and Georgiev 2018; Han et al. 2018a, 2020). The problem is that these built common-sense networks all have their own predefined frame which may not connect two nodes from a design perspective.

Thus, there is an impetus for developing a design-creativity-focused semantic network to meet the growing demands for engineering knowledge discovery, technology information retrieval, engineering design aids and creativity management.However, there are only a few studies focused on constructing semantic networks specifically for engineering design. A creativity-focused semantic network normally builds nodes retrieved from a reliable data source and establishes the association based on statistical or semantic relationship. The statistical relationship that represents the value on associations is assigned with a statistical calculation. For example, Shi et al. (2017) created a large semantic network with statistical relationships in the engineering and design domain. Its statistical relationships are built on the co-occurrence between each pair of words in nearly one million engineering papers and one thousand design posts. He et al. (2019) created a semantic network with a core-periphery structure according to the word clouds embedding co-occurrences information. In this way, the semantic network built the edges on a statistical level and could support engineering and technology creativity from a statistical perspective.

The semantic relationships are the associations that exist between the meanings of words and are applied in many design activities, such as analogy and metaphor methods (Goel 1997; Johnson 1992). For design creativity, Sarica et al. (2019) built a large-scale comprehensive semantic network of technology-related data for engineering knowledge discovery (TechNet). The semantic relationships between words are established by using natural language processing techniques to derive the vector of such terms. Kim and Kim (2012) suggest a cause-and-effect relationship to build a cause-and-effect function network to support technology creativity. With semantic relationships, the network could support data integration, knowledge discovery and in-depth analysis from a semantic perspective (Sarica and Luo 2021; Sarica et al. 2019, 2021).

These two types of relationships own their perspective and benefit, respectively: the statistical relationships could build far-related connections which lead to more creative designs compared with closely related connections (Han et al. 2020); the semantic relationships could present a perspective that analogy and metaphor methods can achieve (Hey et al. 2008; Linsey et al. 2012) and contribute to design creativity by means of semantic association (Casakin and Georgiev 2021). It is expected that a properly combined relationship will take the advantages of both relationships and be beneficial to design activities.

This study plans to build a large encyclopedia-based semantic network with statistical-semantic fused relationships. Inspired by the use of statistical relationships in a semantic network and semantic relationships in the design engineering domain, we aim to build a semantic network that combines the benefits of both the statistical relationship and the semantic relationship to better capture the implicit connection of cross-domain concepts to better stimulate design creativity.

## 3. Construction of WikiLink

In this section, we examine the construction of WikiLink, a semantic network based on Wikipedia data. The Wikipedia items are regarded as the nodes, and the interlinks between the items on the same page are regarded as the directly connected relationship (edges) between nodes. The edges in the network are assigned with a fused weight consisting of two types of weight, and four algorithms are proposed to retrieve relevant knowledge concepts and relationships for design creativity.

### 3.1. Data Source

**Figure 1:**
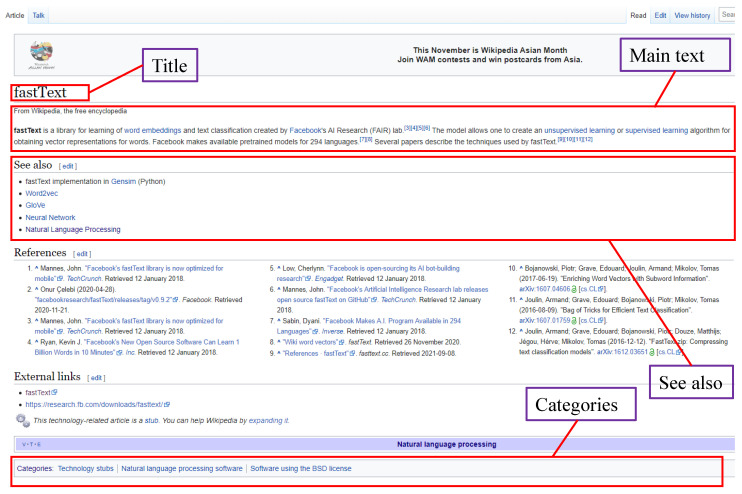
An example page in Wikipedia.

While patents and scientific literature focus on technological and scientific knowledge, an encyclopedia is an integrated source of general knowledge and specific knowledge, with broad coverage of disciplines. Wikipedia, as an online encyclopedia, is unrestricted by the weight and volume and has the potential to be truly comprehensive in knowledge. Wikipedia is written and maintained by a community of volunteers and offers copies of available content to anyone to download. WikiLink processes on English Wikipedia pages before 3 January 2021, comprised 6,408,679 articles. For each Wikipedia article, WikiLink extracts the titles, main text, “see also” and categories for further analysis. Figure 1 is an example page of a Wikipedia article containing a title, main text, “see also” and categories. It should be noted that articles with a colon in the title are excluded. These articles with a colon account for 10% of total articles, which are Wikipedia’s administrative pages and are not relevant as the core source of design information.

### 3.2. Extraction Process

Wikipedia covers 13 main categories to group pages on similar subjects, with each main category having up to 6 layers of subcategories. The deeper the subcategory is, the more specific Wikipedia’s title will be. The articles are firstly filtered based on the indicated categories on their article pages to avoid too specific articles: only the articles within 3-layer subcategories are kept. The network is constructed based on these selected articles’ title, main text and “see also”.

There are two parts in a semantic network: the nodes and relationships between them. The nodes are from three sections in each Wikipedia article: the title, the hyperlinks in the main text and the hyperlinks in the “see also” section. These hyperlinks in the main text are chosen as nodes since they are verified concepts in Wikipedia and indicate explicit associations between concepts as they occur with other concepts in the same articles.

The relationships are assumed to be established between two concepts if they co-occur in the same article. Two different criteria are applied for the raw weight accumulation of each relationship. Since there is a large number of concepts in the main text, if two concepts co-occur in the main text, the weight is assigned a lower value to avoid dominant concepts; the concepts in “see also” are intrinsically strong associations but with less amount compared with the concepts in the main text which are assigned a higher weight. The choice of different weight assignment is determined based on experimental results: if two concepts co-occur in the main text, the weight will be added with one; if two concepts occur in the “see also”, the weight will be added with nine. The raw weight is accumulated and stored for later filtering. In this way, the nodes appearing in one article will be interlinked. Taking the content in Figure 1 as an example, the nodes are “fastText”, “word embeddings”, “Facebook”, “unsupervised learning”, “supervised learning”, “Word2vec”, “Glove”, “Neural Network” and “Natural Language Processing”. The relationships are established between each pair of nodes because they co-occur in the same article. In this way, a network can be constructed by processing all articles in Wikipedia’s database.

### 3.3. Construction of Edge Weights

After the extraction process, an initial network with nodes and edges can be constructed. In the semantic network, explicit knowledge associations are direct edges linking pairs of nodes, and implicit knowledge associations are paths consisting of multiple edges, which means an implicit knowledge association is essentially a concatenation of a series of interconnected explicit knowledge associations (Shi et al. 2017). To evaluate the correlation degree of implicit knowledge associations, the weight of explicit knowledge associations should be quantified.

#### 3.3.1. Semantic Cosine Similarity Weight

In the construction process, the explicit associations are built based on the interlinked concepts within pages, and the corresponding raw weights are statistically calculated. These statistical relationships construct the basic edges in a semantic network from a statistical perspective, which provides the foundation for WikiLink and statistical intuition for information retrieval. In design activities, the semantic relationship also contributes much to design creativity such as analogy and metaphor methods (Hey et al. 2008; Linsey et al. 2012) from a semantic perspective. Inspired by the implication of semantic relationship in design creativity activities, the statistical association between two concepts can be combined and balanced with the semantic similarity for boosting design creativity. The semantic similarity can be obtained by transforming all words to vectors and calculating the semantic cosine similarity between these vectorized concepts. Conventional word embedding methods such as Word2Vec train a unique word embedding for every individual word. However, Wikipedia contains a large number of terms, with some of them even being new terms out of vocabulary. FastText (Bojanowski et al. 2017; Joulin et al. 2016) can solve this issue by treating each word as the aggregation of its subwords. The vector for a word is simply taken to be the sum of all vectors of its component char-ngrams. In this way, fastText can obtain vectors even for out-of-vocabulary (OOV) words, or the new terms in Wikipedia, by summing up vectors for its component char-ngrams, provided that at least one of the char-ngrams was present in the training data. When all concepts have been represented as word vectors, all edges connecting two nodes are assigned with a value by calculating the semantic cosine similarity between these vectors.

#### 3.3.2. Global Normalization and Local Normalization

In many design models, the design creativity process involves two important phases: divergence and convergence (Childs et al. 2022). For example, there are rounds of divergent and convergent phases in the “double diamond” design process model (The Design Council 2017). Divergence is a phase that encourages exploring different solutions as much as possible while convergence follows a particular set of logical steps to arrive at one solution which in some cases is a “correct” solution. Inspired by the principles of divergence and convergence, the retrieval behaviors can be facilitated in two distinct ways: a “general” and “specific” ways. “General” means the nodes are common and basic concepts with a relatively general meaning, which tends to lead divergent thinking in a design creativity process. “Specific” means the nodes are detailed and domain-specific concepts, which have higher potential to guide convergent thinking. “General” and “specific” retrieval are realized by normalizing the raw weight with a globalization method as shown in Equation (1) and a localization method as shown in Equation (2):

(1) $$w_{ij}^{g}=\left(w_{ij}-w_{min}\right)/\left(w_{max}-w_{min}\right)$$

(2) $$w_{ij}^{l}=w_{ij}/S_{i}$$

where *w*_*max*_ and *w*_*min*_ are the maximum and minimum value of the raw weight in the whole network., *w*_*ij*_ is the raw weight between the node *i* and node *j*, and *S*_*i*_ is the sum value of all raw weights of edges around node *i*.

The global normalization performs feature scaling normalization from a global perspective, in which *w*_*ij*_^*g*^ expresses the significance of the strength compared to the whole network. Global normalization tends to retrieve more “general” concepts (Shi et al. 2017). The local normalization performs feature scaling normalization from a local perspective, in which *w*_*ij*_^*i*^ expresses the relative importance of the strength compared to its own adjacent value. Local normalization tends to extract more domain-specific concepts.

#### 3.3.3. Geometric Mean and Harmonic Mean

Since an implicit knowledge association is essentially a concatenation of a series of explicit associations, the accumulation of the strength of contained explicit associations (edges) can potentially indicate the correlation degree of the implicit association (path). Therefore, in order to reflect the overall strength of all the explicit associations in an arbitrary implicit association, the retrieval behaviors can be facilitated in two distinct ways: one type of retrieval, referred to as “basic”, is a short implicit association across fewer edges focusing on relevant concepts which tend to be in the same domain, while another type, referred to as “professional”, is a long implicit association with more edges across multiple distant domains. Therefore, the geometric mean (GM) and the harmonic mean (HM) are applied on the normalized weights for different design creativity behaviors.

The geometric mean (GM) and harmonic mean (HM) are given in Equations (3) and (4), respectively:

(3) $$\mathrm{GM}:w_{\left(k_{1}-k_{2}-\cdots -k_{n+1}\right)}=\sqrt[{n}]{\prod_{k=1}^{n}w_{k,k+1}}$$

(4) $$\mathrm{HM}:w_{\left(k_{1}-k_{2}-\cdots -k_{n+1}\right)}=\frac{n}{\sum_{k=1}^{n}\frac{1}{w_{k,k+1}}}$$

where the *w*_(*k*_1_-*k*_2_-...-*k*_*n*+1_)_ is the overall weight of the path, and *w*_*k,k*+1_ is each weight along the path.

### 3.4. Four Algorithms for Design Creativity

The primary use of the design semantic network is to retrieve relevant knowledge concepts and relationships for design creativity. In addition to retrieving around a single concept, retrieving the implicit associations between two distant knowledge concepts is also introduced. Four algorithms are developed by applying the normalization and mean methods to the proposed retrieval approach. The four algorithms, which are “Explore-General”, “Explore-Specific”, “Search Path-Basic” and “Search Path-Professional” are applied as four functions in WikiLink.

**Figure 2:**
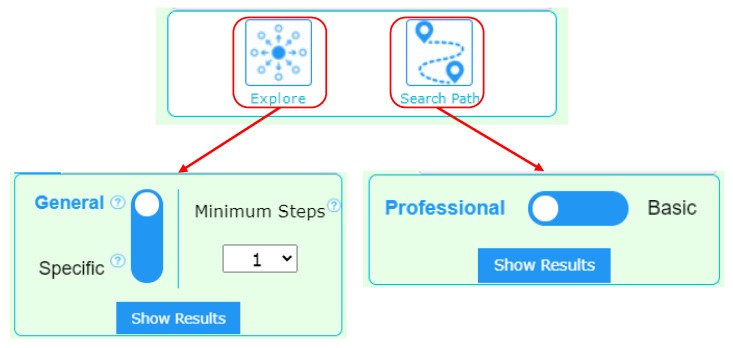
Four functions in the panel of WikiLink.

The “Explore” algorithm is used to explore and retrieve around a single knowledge concept. The retrieved results can be classified as either “general” or “specific”. The “Explore” function panel in WikiLink is shown in Figure 2. Specifically, since it is preferred to retrieve both “general” and “specific” knowledge concepts related to a query, we apply two different normalization algorithms with distinct retrieval behaviours in this “Explore” function. One is global normalization to retrieve “general” concepts for divergence, and the other is local normalization to retrieve “specific” concepts for convergence. The overall weight is calculated on a combination of the statistical weight and the semantic weight. The algorithm for “Explore-General” and “Explore-Specific” are given in Equations (5) and (6), respectively:

(5) $$w_{explore}^{general}=0.3\times \left(1-w_{semantic}^{}\right)+0.7\times w_{}^{g}$$

(6) $$\begin{array}{c} w_{explore}^{specific}=0.2\times \left(1-w_{semantic}^{}\right)+0.8\times w_{}^{l} \end{array}$$

where the *w*_*semantic*_ is the semantic cosine similarity weight, *w*^*g*^ is the statistical weight after global normalization, and *w*^*l*^ is the statistical weight after local normalization.The weights in the algorithm are determined based on experimental results.

The “Explore” algorithms are further combined with the single source Dijkstra’s shortest path algorithm, which starts from the source query to retrieve all reachable nodes in order from the shortest distance. In addition, a “Minimum Step” functionality is provided on the “Explore” panel, where knowledge associations with edges less than the number of the defined minimum step are filtering out for paths with fewer steps. Therefore, the knowledge associations are retrieved and ranked under the combined weight with the minimum step.

The “Search Path” algorithm is used to find implicit associations as paths are given two knowledge concepts. The retrieval result can be classified as either “basic” or “professional”, where “basic” means the path is short and nodes are general concepts, while “professional” means the paths are long and nodes are domain-specific concepts. The “Search Path” function panel in WikiLink is shown on the right side of Figure 2. Specifically, besides two different normalization algorithms, the geometric mean(GM) is further applied to retrieve short implicit associations across fewer edges focusing on relevant knowledge, while harmonic mean(HM) is applied to retrieve long implicit associations with more edges across multiple domains.

The algorithm of “Search Path-Basic” and “Search Path-Professional” are given in Equations (7), (8), (9) and (10), respectively:

(7) $$w_{\left(k_{1}-k_{2}-\cdots -k_{n+1}\right)}^{basic}=\sqrt[{n}]{\prod_{k=1}^{n}w_{k,k+1}^{basic}}$$

(8) $$\begin{array}{c} w_{k,k+1}^{basic}=0.3\times \left(1-w_{semantic}^{}\right)+0.7\times w_{}^{g} \end{array}$$

(9) $$w_{\left(k_{1}-k_{2}-\cdots -k_{n+1}\right)}^{professional}=\frac{n}{\sum_{k=1}^{n}\frac{1}{w_{k,k+1}^{professional}}}$$

(10) $$\begin{array}{c} w_{k,k+1}^{professional}=0.2\times \left(1-w_{semantic}^{}\right)+0.8\times w_{}^{l} \end{array}$$

where the *w*_*semantic*_ is the semantic cosine similarity weight, *w*^*g*^ is the statistical weight after global normalization, and *w*^*l*^ is the statistical weight after local normalization.

## 4. Evaluation

In this section, we conduct four studies on WikiLink to demonstrate its effectiveness and usefulness. Some other semantic networks, which are publicly accepted or aiming for design creativity, are selected as benchmarks during the comparison, including B-link, WordNet, ConceptNet, Wikidata and DBpedia. The evaluation is conducted from four perspectives, i.e., coverage of concepts, coverage of relationships, coverage of disciplines, term-to-term evaluation and effectiveness of combined relationships to provide an overview of the strengths and weaknesses of WikiLink.

### 4.1. Coverage of Golden Concepts

In order to demonstrate the feasibility of WikiLink, golden concepts, which are composed of words and terms, are defined as the benchmark to evaluate WikiLink’s term coverage. To evaluate the coverage in disciplines and ensure the impartiality, the golden concepts should be collected from a data source different from Wikipedia but have the multi-disciplinary structure. The golden concepts are collected manually within an online source Encyclopedia Britannica through several steps. Firstly featured concepts are obtained from its website. There are several categories of topics available concerning different domains, including culture, science and technology. By gathering these classified words and terms, it is ensured that the collected data contains interdisciplinary knowledge. The original data is refined afterward by removing uncommon expressions and standardizing their formats. The aim of this step is to assure the precision of the following evaluations. Eventually, we obtain a list of 468 words and terms, covering knowledge in 8 domains, and part of the concepts are shown in Table 1.

With these golden concepts, we then evaluate how many concepts are contained in WikiLink. The retrieval rate $C_{R}$, as shown in Equation (11), is applied as the metric of concept retrieval:

(11) $$C_{R}=\frac{n_{C}}{N_{C}}$$

where $n_{C}$ means how many concepts are contained in the network, while $N_{C}$ represents the number of golden concepts, which is 468 is this case.

WordNet and ConceptNet are used as two benchmarks for evaluation. DBPedia and other Wikipedia-based network are not assessed since they are all extracted from Wikipedia which will lead to a same result as WikiLink theoretically. It is observed that WordNet only contains 209 concepts, resulting in a low $C_{R}$ rate of .449. The specific $C_{R}$ values of different categories are shown separately in Table 2 (the highest rate is bolded in each line), from which we notice that WikiLink gives the highest retrieval rate, indicating that our network has a wider coverage of concepts compared with the other tools considered.

To be specific, our approach involves more concepts in most categories and achieves the highest retrieval rate. In comparison, WordNet shows overall weaknesses due to its inadequacy in processing two-word terms. ConceptNet has decent performance in the fields of art, science, sports and technology, but it lacks strengths in certain categories such as topics and events.

This result can be explained by the limitation of ConceptNet’s construction properties. Even though the data source of ConceptNet includes two-word terms, such as stained glass, chemical element and mental disorder, these terms are mostly composed of one adjective and one noun. Except for names of countries and regions, seldom are two-noun terms involved in ConceptNet. Based on our observation, plenty of concepts in those two categories, i.e., topics and events, are composed of more than one noun, e.g., teacher education, Paris agreement and Pacific crest trail, which are exactly situations that ConceptNet lacks a solution to. This explains ConceptNet’s low $C_{R}$ rate for those two categories. In contrast, our approach can deal with various kinds of terms, which explains its overall high coverage. This high coverage of concepts can support design creativity with a large concept space.

### 4.2. Coverage of Golden Relationships

A list of golden relationships is selected from the data source as the evaluation benchmark to quantitatively evaluate the performance of relationship coverage. Similar to the construction process of WikiLink, we extracted concept relationships from Encyclopedia Britannica’s spotlight articles. Only those which are composed of golden concepts are retained. We randomly picked 1000 concept pairs from the retained ones and defined as golden relationships.

Denoting golden relationships as set *H*, we compare the performance of WikiLink with other tools in terms of the coverage of golden relationships. In this process, we retrieve all relationships between golden concepts from each tool and denote these retrieved relationships as set *V*. The evaluation metric is defined as follows:

(12) $$\begin{array}{c} \begin{array}{c} R=\frac{\left|V\cap H\right|}{\left|H\right|} \end{array} \end{array}$$

where *R* indicates the retrieving rate of relationships. WordNet, ConceptNet, Wikidata and DBpedia are chosen as benchmarks. The results are shown in Table 3.

Specifically, 15 relationships are retrieved from WordNet, which belong to golden relationships, leading to a significantly low *R* value of only .015. This retrieving rate can be explained by WordNet’s data structure. To our knowledge, WordNet only retrieves specific relationships, including “synonyms”, “sister terms”, “hypernyms” and “hyponyms” between two concepts, which leads to its huge deficiency in context association and results in a low retrieving rate.

The web API of ConceptNet is used to retrieve concepts and relationships. It turns out that there are 170 relationships which are found in the golden relationships, resulting in an *R* value of .170. The retrieving rate can be understood from two perspectives. ConceptNet’s network contains more concepts than WordNet, which can be observed from its $C_{R}$ value. In addition, it provides richer explanations for “relationships”. In other words, as well as “synonyms” and “hypernyms”, ConceptNet is also able to retrieve “related terms” and “terms with this context” for an arbitrary single concept. These two reasons both contribute to its retrieving rate.

Both Wikidata and DBpedia are knowledge graphs based on Wikipedia and can be retrieved with a SPARQL query. It should be noted that in Wikidata, a unique identifier of a concept is required while retrieving the relationships between them. However, some concepts contain multiple semantics which lead to several identifiers. In order to balance the time complexity and performance of retrieval, we sort all the identifiers of each concept in ascending order and take the first three identifiers to form the identifier list of the concept. When retrieving the relationship between two concepts, the identifiers in the two lists are paired, and if any pair exists in the knowledge triple, then this pair of relationships will be considered as covered. Experimental results show that 178 golden relationships are found in Wikidata and 449 in DBpedia. Though DBpedia achieves high coverage since it covers the vast majority of Wikipedia entries, almost half of the golden relationships still cannot be retrieved. The potential reason is that the relationship between two concepts in DBpedia and Wikidata needs to be described with a particular property (e.g., “is instance of”), which means a solid and closely connected relationship and reduces the potential knowledge association for design creativity. For example, “Therapy”, which refers to the means used to solve a health problem, is related to “Public health”, but the relationship between them cannot be described by a particular property. Thus, the golden relationship “Therapy” and “Public health” can be retrieved in Wikilink but not in Wikidata and DBpedia. It should be noted that YAGO is not considered as a benchmark in this evaluation since it is constructed as a knowledge base for real-world named entities, such as person and cities (Pellissier Tanon et al. 2020). The relationships (e.g., “birthPlace”, “ofCountry”) are formed accordingly. These close relationships will not be covered in the golden relationships, and it is unfair to compare with WikiLink.

In the end, 721 relationships can be retrieved from the golden relationships within WikiLink. This can be explained by its largest number of concepts, and the relationships in our approach are defined differently, i.e., they are established between concepts that are shown on the same pages. To summarize, WikiLink achieves a retrieving rate of .721 and shows the best performance. This high retrieving rate of relationships builds enough associations which can potentially contribute to design creativity.

### 4.3. Coverage of Categories

**Figure 3:**
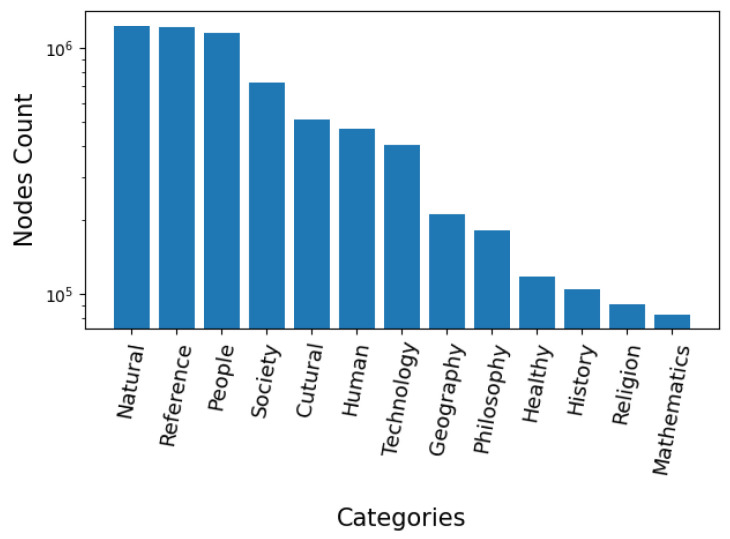
The distribution of concepts in WikiLink.

To demonstrate that WikiLink covers a wide range of categories, we categorize and count all the nodes in WikiLink according to Wikipedia’s category rules. Wikipedia defines 13 main categories: cultural, geography, health, history, human, mathematics, natural, people, philosophy, religion, society, technology and reference. By traversing all the items’ categories in WikiLink, the distribution of the 13 categories is presented in Figure 3.

It can be seen from the graph that WikiLink’s data have a wide distribution among 13 main categories, and the count of a particular main category can reach up to 100,000 nodes. Especially, the natural, people and reference categories have the largest counts, which are 1,241,491, 1,161,583 and 1,222,966, respectively. Rather than focusing only on technological and scientific knowledge, WikiLink is a more generic semantic network, with knowledge from a wide coverage of disciplines, which can be used in daily design creativity activities to obtain inspiration. Specifically, the data source of B-link mainly comes from scientific papers, which leads to the uneven distribution of each discipline, while WikiLink has a wide range of information in different fields and disciplines. Compared with TechNet, the result of WikiLink shows higher diversity as the distribution of TechNet is highly correlated with the distribution of patents, which may affect the inspiration of the design because of the coverage limitation, even though it contains a large number of domains within technology fields.

### 4.4. Term-to-Term Evaluation

To evaluate whether the computed edge weights are consistent with human judgment, thirty term pairs (three groups and each ten as a group) representing various degrees of relevance were prepared by language experts, and ten students were employed to rate the relevance of each pair. The students scored semantic relevance and statistical relevance on a five-scale from one (not related) to five (highly related), and the average of scores is computed for each pair. The semantic relevance and statistical relevance are then combined as the weight in the “Explore-General” algorithm. In this evaluation, only the “Explore-General” edge weights in the four algorithms is evaluated since the weight calculation in the four algorithms is all similar.

With the evaluation results, Cronbach’s alpha is used to measure the inter-rater reliability which is 0.78 as an acceptable result. Spearman’s rank correlation coefficient is then used to assess the relationship between computed edge weights and human judgments. Table 4 shows the result of the Spearman rank correlation coefficients between the pairwise association values of the same term pairs.

The hypothesis of the Spearman correlation coefficient is then tested to determine whether the results are statistically significant. By checking the table of critical values, the three groups’ Spearman’s rho are all greater than the critical value .57 (one tail, α = .05), so the null hypothesis is rejected. This supports that there is a strong correlation between the computed edge weights and human judgments , upheld by a significance level of 95%.

### 4.5. Effectiveness of Combined Relationships

As introduced in Section 3, the statistical relationships between two concepts are established if they co-occur in the same article. Constructing the basic connection from a statistical perspective only could potentially lead to a phenomenon that the retrieval is dominated by some highly common concepts. These dominating common concepts decrease the retrieval probability of other useful concepts for design creativity. However, using semantic relationships only as the weight of edges is beneficial for design but might require longer association for implicit knowledge discovery. The semantic relationships are thus incorporated to balance the statistical relationship. To demonstrate the effectiveness of the proposed weight fusion, three types of retrieval results based on different relationships (networks with combined relationships, with statistical relationships, and with semantic relationships) are compared. The concept “health” is chosen for the “Explore” function, and the concept pair “health and 3d printing” is chosen for the “Search Path” function.

**Figure 4:**
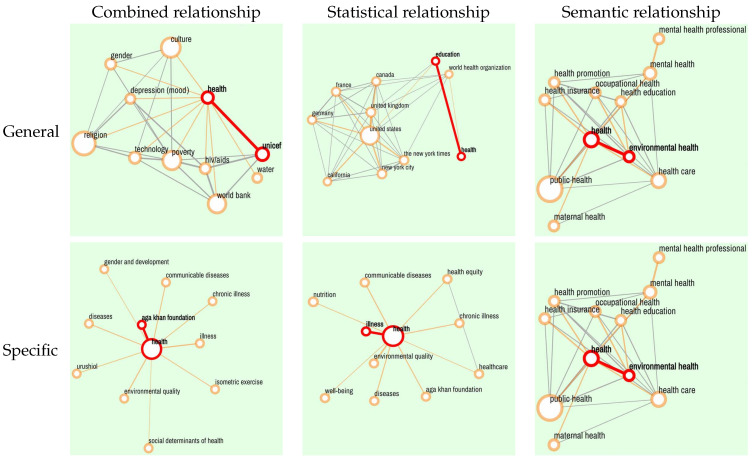
Retrieval results for “health” with three different relationships.

Figure 4 and Table 5 are the results of “Explore” and “Search Path”, respectively. It can be seen that the results of “Explore” and “Search Path” with statistical relationship have more concepts which contain common and general meaning but are irrelevant with “health” semantically, e.g., “United States” and “United Kingdom” which are dominant nodes in this case. Conversely, the results of the two functions with semantic relationships contain more relevant concepts but only show the semantic relevance to “health” (e.g., “environmental health” and “health care”). The combined relationship makes a balance between the statistical relationship and semantic relationship so that it produces a relatively positive result.

The node degree of a concept means the sum of weights of all edges incident to that node. The average node degree of concepts is calculated in combined relationships and statistical relationships to demonstrate whether the very common results are balanced quantitatively. Table 6 and Table 7 show that in four functions the average node degrees of concepts with combined relationship are all observably lower than that of concepts with statistical relationship, which imply that the semantic relationship balances the statistical relationships to retrieve valuable information. Both the quantitative and qualitative results indicate that the combined relationship is efficient to reduce the influence of dominant concepts with high node degree in retrieval results thus facilitating design creativity activities.

## 5. Demonstration

In this section, we showcase four functions in WikiLink for information retrieval and design creativity. Qualitative analysis of the results is performed to demonstrate the features of each function. In addition, the performance of WikiLink is compared with six state-of-the-art tools, and the corresponding results are also analyzed qualitatively.

### 5.1. The “Explore-General” and “Explore-Specific” Mode

**Figure 5:**
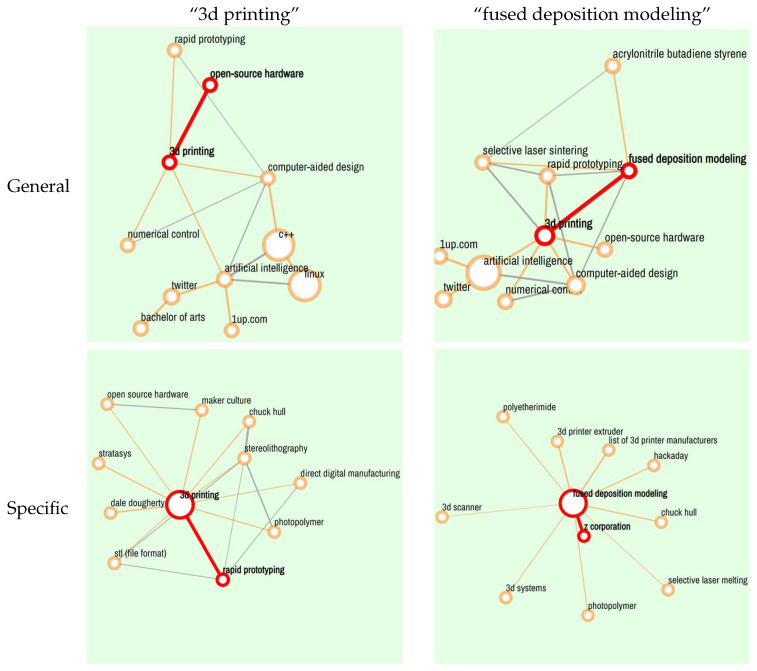
Retrieval results for “3d printing” and “fused deposition modeling” in “Explore-General” and “Explore-Specific” mode.

To fairly compare the performance of “Explore-General” and “Explore-Specific” modes, two terms in the field of engineering design are chosen: “3d printing” and “fused deposition modeling”. 3D printing is a multi-faceted technology, has been employed across a broad range of applications (Berman 2012) and is a widely used term with general meanings. Fused deposition modeling (FDM) is a 3D printing method that heats a continuous thermoplastic filament and extrudes it for layer-by-layer deposition (Hamzah et al. 2018), which is also a widely used term with specific meanings. These two terms are input and explored in “general” and “specific” modes, respectively. Figure 5 shows the top 10 relevant terms in each retrieval. By comparing the “general” results (the first row) with the “specific” results (the second row), it can be seen that the terms in “general” results are more common and comprehensible, such as computer-aided design and artificial intelligence, while the terms in “specific” results, such as stl (file format) and polyetherimide, are normally very specific concepts in particular domains. Furthermore, as the figure shows, FDM’s specific result is centered scattering. This implies that primary terms in a particular domain are discrete and irrelevant to each other.

### 5.2. The “Search Path-Basic” and “Search Path-Professional” Mode

The “Search Path” function allows users to explore the implicit associations between two items even from different domains. It also has two modes that can return two types of associations. In order to test the above two modes, we used two pairs of terms, “brain” and “computer”, which are weakly related, and “avocado” and “chair”, which are seemingly unrelated. Table 8 shows the retrieved highest-correlated “basic” and “professional” knowledge associations of the two pairs. Obviously, the “basic” paths are shorter, and the “professional” paths are longer. Most of the nodes in “basic” paths are concepts with general meanings between the two domains, such as artificial intelligence, fruit and furniture, while the “professional” path is longer and the nodes are almost scientific terms or specific objects such as “neuroscience”, “xylitol” and “guacamole”. Some explicit associations are discovered in the results. For example, brain science drives the advance of computer science, especially artificial intelligence, which appears in the path “brain → artificial intelligence → computer”. In addition, more implicit associations are connected by some surprising concepts, such as “fruit”, “furniture” and “rocking chair”, which may provoke the idea of fruit-shaped furniture, such as an avocado-shaped rocking chair. It is found that in some cases purely statistical weights between edges result in a longer and more surprising path which may inspire more innovative ideas in design activities.

### 5.3. The “Explore” and “Search Path” Function

**Figure 6:**
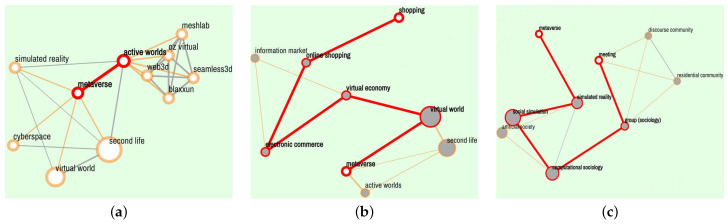
Comparisons of the results from “Explore” and “Search Path”: (**a**) the “Explore Specific” results for “metaverse”; (**b**) the “Search Path-Professional” results between “metaverse” and “shopping”; (**c**) The “Search Path-Professional” results between “metaverse” and “meeting”.

The above shows that the “Explore” function aims to discover the knowledge associations around a single term, while the “Search Path” function aims to search for the associations between two terms. To clarify the difference between them, a hot concept in engineering design, “metaverse”, was explored along with two weakly related terms separately: “shopping” and “meeting”. Retrieval experiments were conducted in “Explore-Specific” and “Search Path-Professional” mode, respectively. As shown in Figure 6, the retrieval results of “metaverse” cover a wide range of fields, including “virtual world”, “simulated reality”, “cyberspace” and related games, including “Second Life” and “Active Worlds”. These wide results can lead to comprehensive knowledge discovery and an open imagination about the target term. On the other hand, the paths between “metaverse” and a selected concept focus on bridging the fields that connect them, which leads to combinational ideas. For instance, the nodes linking “metaverse” and “shopping” are related to “virtual economy”, and the nodes linked “metaverse” and “meeting” are related to virtual society.

### 5.4. Comparison with Benchmark Tools

We undertook a retrieval comparison between WikiLink and the other four benchmark tools. The target terms are “neural network” in computer science and “trypsin” in medical physiology. This experiment aims to test whether our network can return a broad range of related terms which can stimulate creativity in the design process efficiently. Since the number and presentation of retrieval results vary from tool to tool, we selected the top 10 related terms for each tool to present in Table 9. Especially, the results of WikiLink and B-link (Shi et al. 2017) were obtained through their “Explore” function. The result of ConceptNet (Speer et al. 2017) was obtained from its “Related terms” category. The result of WordNet (Fellbaum 2010) was obtained from its “Synset” and “Example sentence” functions. The result of Wikidata was obtained from its “Search Wikidata” function, and since the term “neural network” in Wikidata refers specifically to the structure in biology, the search for this was redirected to the term “artificial neural network” in machine learning which more closely matches our expectations. The result of DBpedia was obtained from its “Keyword Search” function. In addition, we have also tried to compare with the large knowledge base “YAGO”, but the retrieval results are mostly translations of target terms in other languages (e.g., “Trypsiini” in Finish is obtained when searching for “Trypsin”), which does not make sense for stimulating creativity in the design process. The main reason is that YAGO is a knowledge base focusing on real-world named entities, such as people, cities and organizations, but is not suitable for the terms with semantics (Pellissier Tanon et al. 2020). Therefore, YAGO was not included in our comparative experiments.

According to Table 9, the terms retrieved by WikiLink in the “general” and “specific” modes both demonstrate the effectiveness of the “Explore” function. For example, the retrieval results of “neural network” in the “specific” mode are all domain-specific terms related to the components (e.g., “artificial neuron”), functions (e.g., “cognitive model”) and applications (e.g., “deep learning”) of “neural network”. Since the “Explore” function of WikiLink is divided into the “Explore-General” and “Explore-Specific” modes, its results, containing common terms (from the “Explore-General” mode) and technical terms (from the “Explore-Specific” mode), cover a comprehensive range. In contrast, ConceptNet, WordNet, TechNet, Wikidata and DBpedia simply have only one retrieval mode, which leads to their retrieved results invariably focusing on some technical terms in a specific range. Furthermore, the retrieval result of “trypsin” from DBpedia contains some terms that are only lexically similar but semantically irrelevant (e.g., “Nybergsund IL-Trysil” and “Trysil”), which may mislead the user’s understanding of the target term. Even though B-Link retrieves in the two modes as WikiLink, its results are also limited by the data source which is engineering academic papers and design websites. It can be seen that the retrieved terms of B-Link tend to contain specific meanings. Instead, WikiLink applies Wikipedia as the data source for its semantic network, which covers information from a wide range of domains. The comparison suggests that WikiLink is more capable of retrieving terms in various domains, which is essential for knowledge discovery in the knowledge-intensive design creativity process.

### 5.5. A Design Case

**Figure 7:**
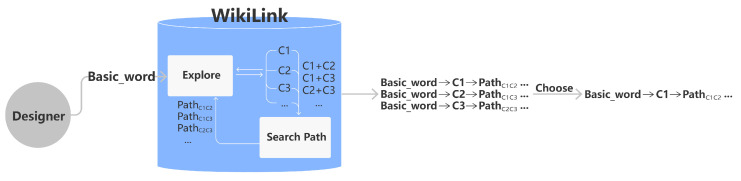
The flow of concepts exploration in WikiLink.

A designer was recruited to conduct a design case and demonstrate the process of applying WikiLink for design creativity. Generally, the designer would be initially given a design question with a “Basic word” and then required to apply the “Explore” function and “Search Path” function in WikiLink to freely explore the related concepts around the “Basic word” which could potentially inspire the designer. By applying the “Explore function”, the designer could discover the knowledge concepts “C1”, “C2” and “C3” around the “Basic word” as denoted in Figure 7. The “Search Path” function provides the paths, e.g., *Path*_*C1C2*_ between two terms “C1” and “C2” for combinational creativity (Han et al. 2019). This process can be iteratively applied to discover knowledge associations and paths such as “C3” and *Path*_*C1C3*_. The related concepts obtained from WikiLink are then used to form design inspiration links such as “Basic_word-C1, *Path*_*C1C3*_, *Path*_*C2C3*_”, and some of them are eventually chosen for the design output of specific design ideas.

**Figure 8:**
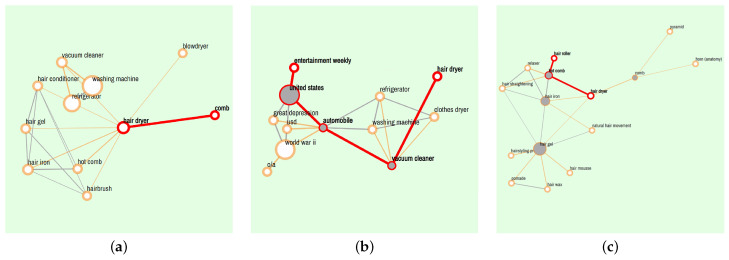
The examples of concepts retrieved by “Explore”: (**a**) “Explore-General” results with one step for “hair dryer”; (**b**) “Explore-General” results with two steps for “hair dryer”; (**c**) “Explore-Specific” results with two steps for “hair dryer”.

A real design case is conducted to illustrate how to facilitate design creativity with WikiLink. Since a “hair dryer” is a well-known product archetype with homogenization in the market and its creativity has encountered a bottleneck, the designer is required to generate ideas and provide innovative designs for a hair dryer. The concept “hair dryer” is chosen as the design query (also known as the basic word) in WikiLink in this case. The designer then starts with the “Explore” function by freely choosing several different step lengths and switching between general and specific mode for divergent and convergent thinking. Some screenshot examples are shown in Figure 8. It is noted that the designer is not restricted to using “hair dryer” as the only query. After the initial exploration in WikiLink, the designer obtained some interesting and inspiring concepts, such as “Entertainment weekly”, “Vacuum cleaner”, “Comb”, “Hair iron”, “Hair gel”, “Hair roller”, “Hot comb”, “Horn” and “Pyramid”. The next step is to apply the “Search Path” function by freely querying the paths between two concepts of the designer’s interests. Some retrieval results are shown in Table 10.

**Figure 9:**
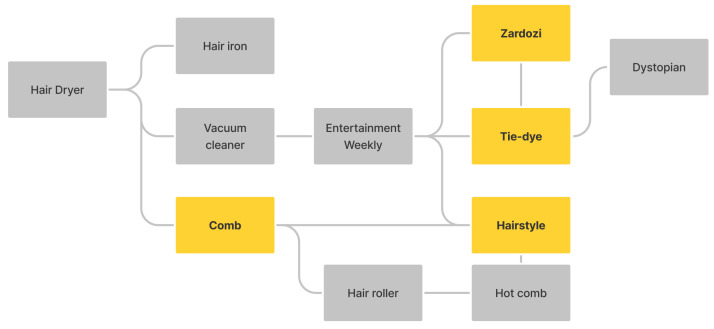
The example of “design inspiration link”.

The designer continued to explore knowledge concepts for design creativity stimuli by iteratively using the “Explore” and “Search Path” functions. The “Explore” function helps discover the knowledge associations around a single term, while the “Search Path” function can potentially look for the associations between two terms. The designer recorded all the interesting and inspiring concepts and formed the “design inspiration links”, as shown in Figure 9, where the base of the link is “Hair Dryer”, and rest of the concepts were from WikiLink obtained by using “Explore” and “Search Path” functions. The above process was repeated to produce at least one design inspiration link and until the designer thought it is enough to formulate design ideas. Eventually, with the ideas originating from the concepts in the inspiration link, the designer produced the final complete design scheme and drew corresponding design sketches.

**Figure 10:**
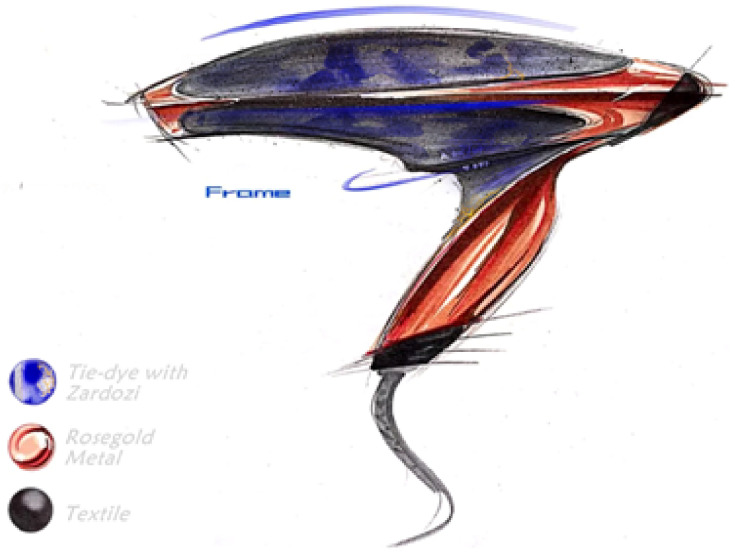
The sketch of the hair dryer inspired by “Tie-dye” and “Zardozi”.

**Figure 11:**
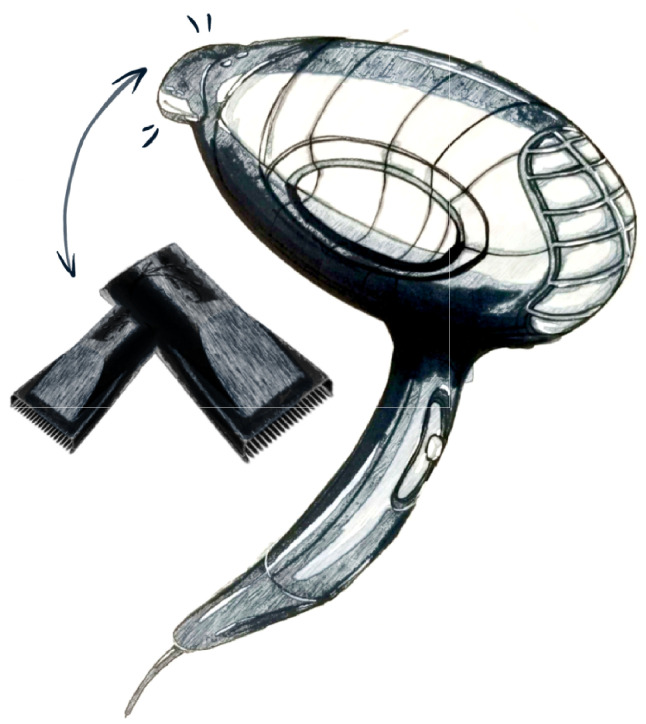
The sketch of hair dryer inspired by “Comb”.

In particular, we use Figure 10 and Figure 11 as the designs produced with the inspiration links “Hair dryer” , “Comb”, “Hairstyle”, “Tie-dye”, “Zardozi”.

In particular, two ideas were generated during the designer’s manipulation with WikiLink. The first design, as shown in Figure 10, is in appearance a design inspired by “Tie-dye” and “Zardozi”. Existing hair dryers in the market are mostly a single pure color with a smooth or frosted plastic shell. “Tie-dye”, the characteristic of the Bai nationality, has special patterns which are uneven in-depth and rich in layers, and overcomes the rigidity of pure color. “Zardozi”, a traditional Chinese craft, has a delicate touch feeling compared with plastic material. Thus “Tie-dye” and “Zardozi” inspire the designer to integrate traditional Chinese cultural elements into the design of a hair dryer to increase cultural connotation. The second design (Figure 11) is functional and inspired by “Hairstyle” and “Comb” in the design inspiration link. The idea is to design the replaceable hair dryer nozzle with the features of “Comb” so that users can comb their hair conveniently while drying the hair without searching it in a hurry.

## 6. Conclusions

A semantic network for design creativity has been constructed. Wikipedia is applied as the data source for the semantic network. During the construction, the Wikipedia items are regarded as the nodes, the interlinks between the items on the same page are regarded as the directly connected relationship (edges) between nodes. The evaluation result indicates that the network contains information from a wide range of fields and expands the data to a new boundary. Instead of simply one type of weight, a combined weight is introduced for the relationship in the semantic network. The combined weight fuses the statistical relationship and semantic relationship which better captures the implicit connection between concepts for design creativity. Four algorithms have been developed to retrieve relevant knowledge concepts and relationships with different levels and manners. The constructed semantic network for design creativity is further developed as a tool, called WikiLink. An evaluation and demonstration for WikiLink were conducted. Compared with other benchmarks, with the fusion of semantic meaning weight and statistical weight, WikiLink can well balance the breadth and depth in exploring knowledge for design creativity. A design case was conducted to demonstrate the process of how WikiLink can facilitate idea generation. The results indicate that WikiLink can serve as a design ideation tool for design creativity.

The study leaves space for future research although it does provide a functional panel for practical use. The weight strength fusing two types of weight is one of the main contributions in this research, but it only shows the numerical value and lacks explicit semantic meaning describing the relationship between two concepts. Thus, a semantic description is expected to be added to the edges in WikiLink and provide richer information for design creativity. The network visualization of WikiLink is currently designed on a two-dimensional scale, which might cause an information explosion when the retrieved network keeps growing. A three-dimensional scale network along with other information visualization techniques could be a solution and provide a more dynamic way for users to explore information and obtain inspiration more effectively.

## Figures and Tables

**Table 1:** The overview of golden concepts.

Categories	Related Concepts
Animal	bird, chordate, coral, insect, sea otter, ...
Art	acting, ballade, chinese literature, emmy award, film, ...
Event	American civil war, bronze age, cold war, French revolution, hurricane Katrina, ...
Place	Africa, Anatolia, Berlin, Cape Town, Indonesia, ...
Plant	carnivorous plant, venus flytrap, ...
Science	atmosphere, brain, carbohydrate, chemistry, disease, ....
Sports	athletics, boxing, gymnastics, rugby, ...
Technology	airplane, bicycle, industry, radar, smartphone, supercomputer, ...
Topic	accident, architecture, buddhism, cbs corporation, democracy, ...

**Table 2:** Retrieving results of golden concepts.

Categories	WordNet	ConceptNet	WikiLink
Total Rate CR	.449	.810	**.938**
art	.386	.818	**.841**
animal	**1.000**	**1.000**	**1.000**
event	.037	.630	**.963**
place	.602	**1.000**	**1.000**
plant	.333	**1.000**	**1.000**
science	.631	**.954**	**.954**
sports	.652	**.957**	.913
technology	.636	.818	**.909**
topic	.287	.638	**.920**

**Table 3:** Evaluation results of golden relationships.

Categories	Count	R
WordNet	15	.015
ConceptNet	170	.170
Wikidata	178	.178
DBpedia	449	.449
WikiLink	721	.721

**Table 4:** Term-to-term evaluation results.

Group Number	Spearman Correlation
1	.69
2	.89
3	.64

**Table 5:** The high-correlated knowledge associations between “health” and “3d printing” with three different relationships.

	Combined Relationship	Statistical Relationship	Semantic Relationship
Basic	health → economics → Massachusetts Institute of Technology → 3D printing	health → education → United States → The New York Times → artificial intelligence → 3D printing	health → health care → palliative care → intensive care unit → 3D printing
Professional	health → construction → ladder → 3D printing	health → physical fitness → physical strength → eccentric contraction → weight plate → knurling → deep drawing → hydroforming → direct metal laser sintering → rapid prototyping → 3D printing	health → health care → palliative care → intensive care unit → 3D printing

**Table 6:** The average node degree of retrieval results for “health” with two different relationships.

Category	“Explore-General”	“Explore-Specific”
Statistical relationship	536	63
Combined relationship	308	32

**Table 7:** The average node degree of knowledge associations between “health” and “3d printing” with two different relationships.

Category	“Search Path-Basic”	“Search Path-Professional”
Statistical relationship	565	139
Combined relationship	473	131

**Table 8:** The high-correlated two types of knowledge associations.

	Brain and Computer	Avocado and Chair
Basic	brain → artificial intelligence → computer	avocado → fruit → furniture → chair
	brain → biology → computer	avocado → walnut → furniture → chair
Professional	brain → neuroscience → psychology → science → technology → computer	avocado → guacamole → burrito → xylitol → product call → ikea → rocking chair → chair
	brain → neuroscience → psychology → science → technology → Internet → computer	avocado → guacamole → taco → hockey puck → potato chips → ladder → rocking chair → chair

**Table 9:** The top 10 related terms to “neural network” and “trypsin” in WikiLink and the 4 benchmark tools.

	Neural Network	Trypsin
WikiLink (general)	deep learning, google, c++, linux, cross-platform, javascript, open-source software, operating system, perl	amino acid, pancreas, enzyme, transcription (genetics), translation (genetics), base pair (genetics), life, active site, translation (biology), stroke
WikiLink (specific)	classification rule, deep learning, cognitive model, stockfish (chess), machine learning, black box, Hebbian learning, list of memory biases, deepmind, artificial neuron	phenylisothiocyanate, myotoxin, triosephosphateisomerase, zymogen, tandem mass spectrometry, peptide mass fingerprinting, ligase, dihydrofolate reductase, pepsin, papain
B-link (general)	genetic algorithm, optimization, fuzzy logic, classification, pattern recognition, artificial neural network, multi-objective optimization, simulated annealing, simulation, response surface methodology	chymotrypsin, protease, pepsin, purification, thrombin, digestion, characterization, expression, synthesis, crystal structure
B-link (specific)	backpropagation, genetic algorithm, fuzzy logic, self-organizing map, multilayer perceptron, backpropagation algorithm, neuro-fuzzy, pattern recognition, neuro-fuzzy system, artificial intelligence	chymotrypsin, enzyme thermostability, modified enzyme, pepsin, protease-activated receptor-2, protease-activated receptor, digestive protease, pyloric caecum, carboxypeptidase a, viscera
ConceptNet	neural net; autoencoder, backpropagation, catastrophic interference, computational intelligence, condela, convolutional neural network, dropout, hidden layer	antitrypsin, antitryptic, apronitin, chymotrypsin, endopeptidase, enterokinase, meromyosin, mesotrypsin, ovoinhibitor, ovomucin
WordNet	neural net, computer architecture, network of neurons, network of nuclei	enzyme, pancreas, protein, polypeptide units
TechNet	artificial neural network, machine learning, training data, pattern recognition, hidden layer, layer node, upper hidden layer, neuron, residual activation, automobile overspeed, vehicular safety sensor, time many	proteolytic enzyme, pepsin trypsin, subtilisin family, bromelain ficin, proteolytic, no amidolytic, enzymatic, amidolytic, protease, trypsin thrombin plasmin, dynorphin targeting moiety, irtx
Wikidata	artificial intelligence, machine learning, discriminative model, types of artificial neural networks, biological neural network, activation function, neuron layer, loss function, optimizer	serine endopeptidase, digestive enzyme, serine-type endopeptidase activity, enzymes, Armenian Soviet Encyclopedia
DBpedia	artificial neural network, convolutional neural network, recurrent neural network, neural network software, physical neural network, feedforward neural network, neural circuit, quantum neural network, network: computation in neural systems, types of artificial neural networks	trypsin inhibitor, aprotinin, trypsin 1, nybergsund il-trysil, prss2, ulinastatin, alpha-1 antitrypsin, trysil, calicivirin, alpha-1 antitrypsin deficiency

**Table 10:** The paths between the inspiring concepts and “hair dryer” retrieved by “Search Path”.

Query Concepts	Mode	Retrieval Results
hair dryer and entertainment weekly	Basic	hair dryer → vacuum cleaner → automobile → united states → entertainment weekly
hair dryer and entertainment weekly	Professional	hair dryer → hair iron → natural hair movement → afro → tie-dye → zardozi → choli → crop top → the face (magazine) → arena (magazine) → loaded (magazine) → fhm’s 100 sexiest women (uk) → fhm → maxim (magazine) → people (magazine) → entertainment weekly
hair dryer and tie-dye	Basic	hair dryer → vacuum cleaner → automobile → textile → tie-dye

## Data Availability

The code and data are published on https://github.com/zjud3/WikiLink, accessed on 2 November 2022.
